# Supplementary material for: Characteristics of Classical Swine Fever Virus Variants Derived from Live Attenuated GPE− Vaccine Seed
Source: Viruses. 2021 Aug 23;13(8):1672. doi: 10.3390/v13081672 (PMC8402697; doi:10.3390/v13081672)
Supplement: Supplementary file 1 [file viruses-13-01672-s001.zip › viruses-1334343-supplementary.pdf]

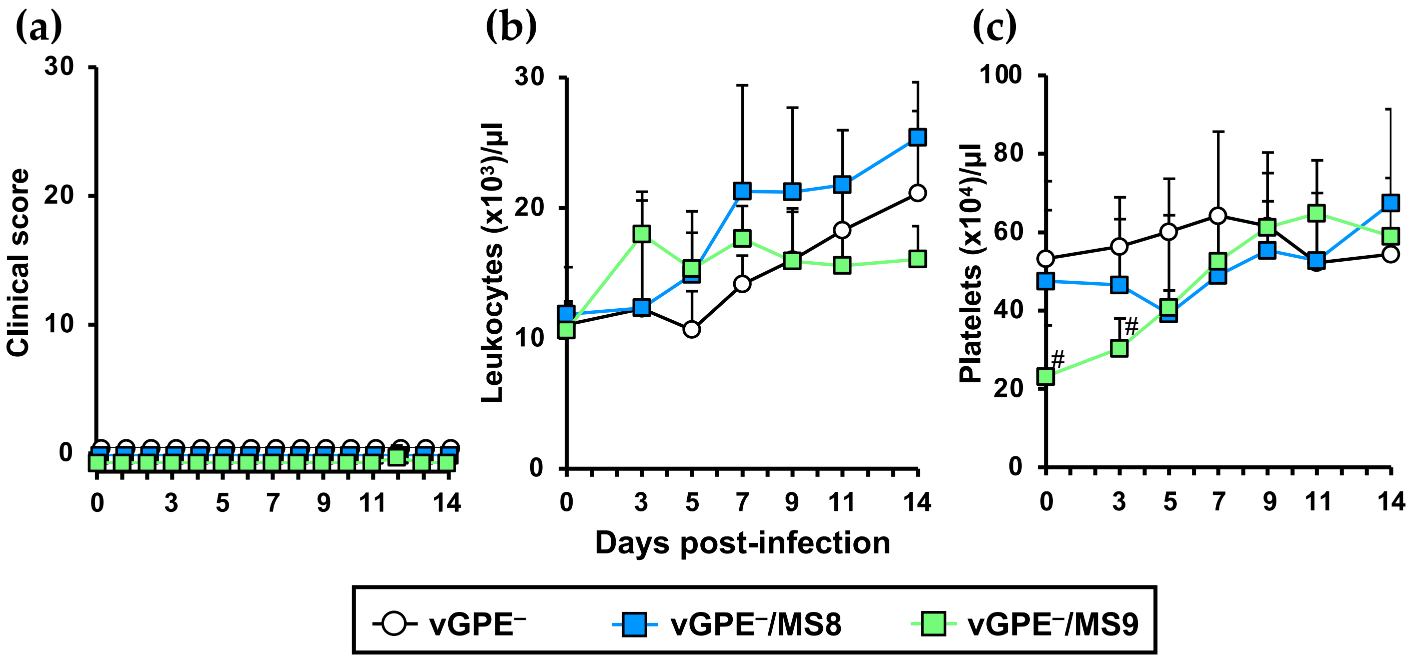

Figure S1. Clinical scores, leukocyte and platelet counts of pigs infected with vGPE⁻ and seed variants. Groups of 5 pigs were infected with the indicated viruses, and blood was collected on 0, 3, 5, 7, 9, 11, and 14 dpi. (**a**) the clinical scores were daily monitored; (**b** and **c**) the leukocyte and platelet counts were measured at each time point. All objects are shown as mean values, with error bars representing the standard deviations. The significance of differences was calculated using one-way ANOVA followed by Student's *t*-test with Bonferroni correction. # indicates $p < 0.025$ between vGPE⁻ and vGPE⁻/MS9.

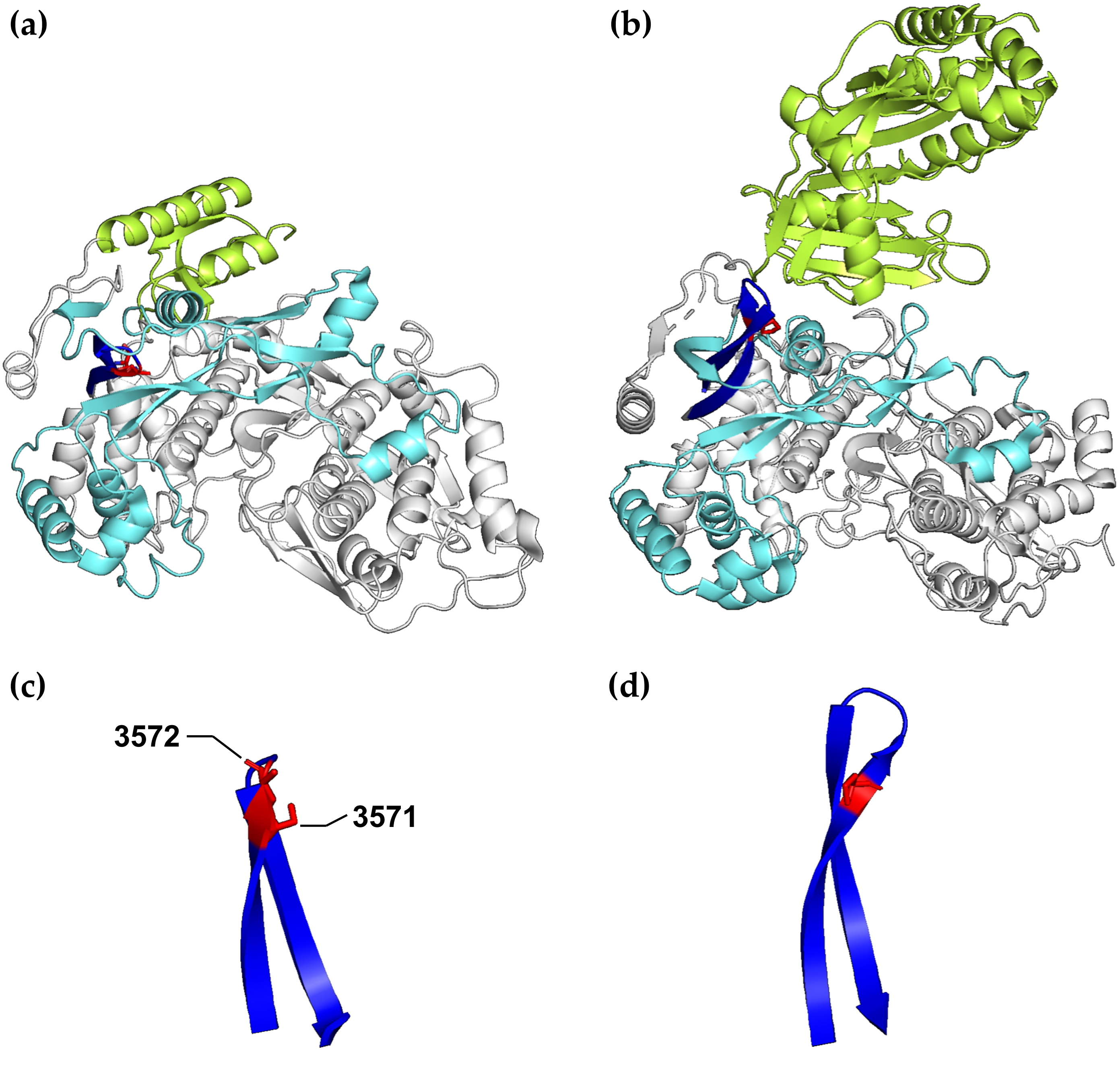

Figure S2. Conformational similarity of CSFV NS5B and JEV NS5. (**a**) the crystal structure of CSFV NS5B (PDB#5Y6R) is shown. The NTD (light green), the middle finger (blue), and the finger domain (light blue) are colored. Amino acids at the positions of 3571 and 3572 are colored in red; (**b**) the crystal structure of JEV NS5 (PDB#4K6M) is shown. The MTase (light green), the middle finger (blue), and the finger domain (light blue) are colored. A key amino acid for the interaction with MTase is colored in red; (**c**) only the middle finger of CSFV NS5B was abstracted; (**d**) only the middle finger of JEV NS5 was abstracted.
